# Diagnostic value of dual-layer spectral detector CT in differentiating lung adenocarcinoma from squamous cell carcinoma

**DOI:** 10.3389/fonc.2022.868216

**Published:** 2022-12-02

**Authors:** Ronghua Mu, Zhuoni Meng, Zixuan Guo, Xiaoyan Qin, Guangyi Huang, Xuri Yang, Hui Jin, Peng Yang, Meimei Deng, Xiaodi Zhang, Xiqi Zhu

**Affiliations:** ^1^ Department of Radiology, Graduate School of Guilin Medical University, Guilin, China; ^2^ Department of Radiology, Nanxishan Hospital of Guangxi Zhuang Autonomous Region, Guilin, China; ^3^ Philips (China) Investment Co., Ltd., Chengdu Branch, Chengdu, China

**Keywords:** lung cancer, pathological classification, dual layer detector, energy spectrum, X-ray computed tomography

## Abstract

**Background and objective:**

The pathological type of non–small cell lung cancer is considered to be an important factor affecting the treatment and prognosis. The purpose of this study was to investigate the diagnostic value of spectral parameters of dual-layer spectral detector computed tomography (DLCT) in determining efficacy to distinguish adenocarcinoma (AC) and squamous cell carcinoma (SC), and their combined diagnostic efficacy was also analyzed.

**Methods:**

This is a single-center prospective study, and we collected 70 patients with lung SC and 127 patients with lung AC confirmed by histopathological examination. Morphological parameters, plain scan CT value, biphasic enhanced CT value, and spectral parameters were calculated. The diagnostic efficiency of morphological parameters, spectral parameters, and spectral parameters combined with morphological parameters was obtained by statistical analysis.

**Results:**

In univariate analysis, seven morphological CT features differed significantly between SC and AC: tumor location (distribution), lobulation, spicule, air bronchogram, vacuole sign, lung atelectasis and/or obstructive pneumonia, and vascular involvement (all *p* < 0.05). In the arterial phase and the venous phase, the spectral parameters of AC were higher than those of SC (AP-Zeff: 8.07 ± 0.23 vs. 7.85 ± 0.16; AP-ID: 1.41 ± 0.47 vs. 0.94 ± 0.28; AP-NID: 0.13 ± 0.04 vs. 0.09 ± 0.03; AP-λ: 3.42 ± 1.10 vs. 2.33 ± 0.96; VP-Zeff: 8.26 ± 0.23 vs. 7.96 ± 0.16; VP-ID: 1.18 ± 0.51 vs. 1.16 ± 0.30; VP-NID: 0.39 ± 0.13 vs. 0.29 ± 0.08; VP-λ: 4.42 ± 1.28 vs. 2.85 ± 0.72; *p* < 0.001). When conducting multivariate analysis combining CT features and DLCT parameters with the best diagnostic efficacy, the independent predictors of AC were distribution on peripheral (OR, 4.370; 95% CI, 1.485–12.859; p = 0.007), presence of air bronchogram (OR, 5.339; 95% CI, 1.729–16.484; p = 0.004), and presence of vacuole sign ( OR, 7.330; 95% CI, 1.030–52.184; p = 0.047). Receiver operating characteristic curves of the SC and AC showed that VP-λ had the best diagnostic performance, with an area under the curve (AUC) of 0.864 and sensitivity and specificity rates of 85.8% and 74.3%, respectively; the AUC was increased to 0.946 when morphological parameters were combined, and sensitivity and specificity rates were 89.8% and 87.1%, respectively.

**Conclusion:**

The quantitative parameters of the DLCT spectrum are of great value in the diagnosis of SC and AC, and the combination of morphological parameters and spectral parameters is helpful to distinguish SC from AC.

## Introduction

Lung cancer is a disease that seriously affects human health. Approximately 85% of patients are non–small cell lung cancer (NSCLC), of which adenocarcinoma (AC) and squamous cell carcinoma (SC) are the most common subtypes (1, 2). Histological classification of lung cancer has been demonstrated as an independent prognostic indicator. Because there are significant differences in biological behavior, treatment strategies and prognostic evaluation between different pathological subtypes of lung cancer (3, 4), a reasonable choice of treatment strategies can reduce mortality, prolong the survival time, and improve the quality of life.

Chest CT is the preferred imaging examination for the diagnosis of lung cancer. Traditional CT can evaluate the benign and malignant lesions according to their morphological features, intensity, and lymph node metastasis. However, it is difficult to evaluate the pathological subtypes of lung cancer because of the lack of quantitative indicators. If morphological criteria did not help to distinguish benign from malignant lung lesions, then it is strongly dependent on invasive pathologic examination or follow-up studies (5). As a non-invasive and effective pathological classification method of lung cancer, energy spectrum CT has become a hot spot in clinical research. The basic structure of the new generation of spectral CT is similar to that of ordinary CT, but it has two spatially equivalent detectors: the upper layer only absorbs low-energy photons, whereas the lower layer absorbs high-energy photons. On the premise of a perfect match in time and space within the projected data domain, high-energy and low-energy data can be parsed to obtain both traditional CT images and spectral images (6, 7).

Previous studies on energy spectrum CT in SC and AC are insufficient and controversial. Fehrenbach et al. (8) considered that only normalized iodine density (NID) in the arterial phase (AP) was significant for the differential diagnosis of NSCLC. Wang et al. (9) showed that the slope of the spectral curve (λ) of 40 to 70 keV and iodine density (ID) in AP had diagnostic value in differentiating SC from AC, but the venous phase (VP) parameters were not included in the study. In contrast, Li et al. (10) showed that VP-ID can differentiate SC from AC by reflecting tumor microvessel density. Jia et al. (11) showed that AP-λ (40–100), AP-Zeff, AP-ID, and VP-ID were significant for their identification, but these data of SC were greater than that of AC. Problems, such as single-phase scanning, single spectral parameters, and the exclusion of morphological parameters, have caused a lot of controversies.

This study improves the above issues. The purpose of this study was to investigate the diagnostic value of spectral parameters of DLCT in determining efficacy to distinguish AC and SC, and their combined diagnostic efficacy was also analyzed.

## Materials and methods

### Patients

**Figure 1:**
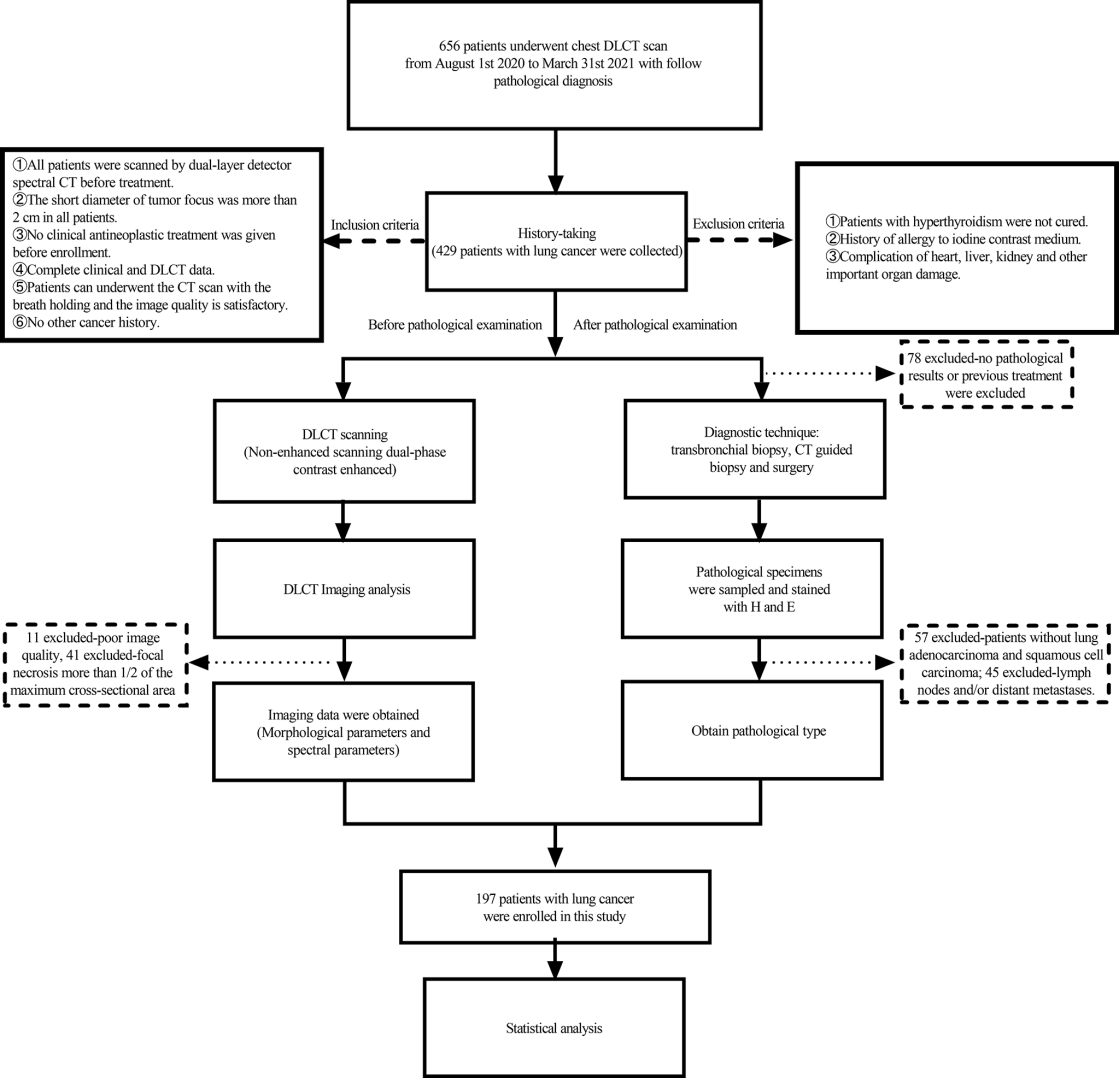
Study flowchart for the inclusion and exclusion criteria of the lung cancer patient sample.

A prospective study was conducted in our hospital between 1 August 2020 and 31 March 2021; a total of 656 consecutive patients who were diagnosed with lung cancer, confirmed by histopathological examination, were enrolled for the present study. To ensure the reliability of the results, all patients were required to conduct DLCT scan within 7 days before treatment. Inclusion criteria were as follows: (1) patients who underwent dual-layer detector spectral CT before treatment; (2) imaging, clinical, and pathological data were complete; (3) the short diameter of the tumor was greater than 2.0 cm; (4) no clinical antineoplastic treatment was given before enrollment; (5) patients can undergo the CT scan with the breath-holding; and (6) no other cancer history. Exclusion criteria were as follows: (1) patients with hyperthyroidism were not cured; (2) history of allergy to iodine contrast medium; and (3) complication of the heart, liver, kidney, and other important organ damage. The flow diagram of the present study is shown in **Figure 1**.

This single-center prospective study was approved by the Hospital Institution Review Committee [no. 2020(KY-E-30)], and written informed consent was obtained from all of the participants.

### CT examination

All scans were performed using the IQon Spectral CT device (Philips Healthcare). The scans included routine CT plain film and AP and VP enhancement scan of the lung. The field of vision ranges from apex to diaphragm level. The contrast agent iophorol (70 ml of iopromide; Beijing Beilu Pharmaceutical Co., Ltd.) was administered *via* an antecubital vein at an intended flow rate of 3.0 ml/s with a high-pressure syringe, followed by 30 ml of saline, injected at the same flow rate. Scans were performed using the bolus chase method. The region of interest (ROI) was located in the descending aorta with a trigger threshold of 150 Hu. AP was started at 6 s after contrast agent injection, and VP was performed at 36 s after contrast agent injection. Parameters included tube voltages of 120 kVp, spectral CT adaptive current, collimator width of 64 × 0.625 mm, pitch of 1.234, rotation time of 0.27 s, and matrix of 512 × 512. After the scans were completed, the data obtained in the enhanced double phases were reconstructed by projected spatial-spectral reconstruction (spectral, level 4). The image reconstruction image thickness was 1 mm, and the image spacing was 1 mm.

### Imaging analysis

The images were sent to the Philips Spectral Diagnostic Suite 9.0 (Philips Healthcare) workstation, and all data were processed and analyzed by two radiologists with more than 5 years of chest CT diagnosis experience. They were blinded to any patient’s clinical data to mitigating potential cognitive biases. Three consecutive image sections containing the maximum cross section of the tumor and the adjacent upper and lower levels were chosen for measurement; the round or oval ROI was drawn as large as possible (areas close to half to two-thirds of the lesion area) to minimize the influences of noise and the partial volume effect. Two radiologists first sketched the ROI on the conventional CT enhanced and unenhanced images, and the morphological parameters (including location, diameter, margin, internal, and other features) of the tumor were evaluated. The ROI was placed on solid regions of the tumor, avoiding areas with vessels, calcification, cystic, and necrotic change. They then obtained data from other parameter images [spectral CT-mono energetic (40 and 60 keV), Z effective (Zeff) images, ID images, etc.], and copy/paste functions were used to ensure the consistency of the size, shape, and position of the ROIs. Enhancement value was defined as the difference of the CT value (AP/VP) and plain CT value according to the following formula: enhancement value (AP/VP) = CT value (AP/VP) − plain CT value. To minimize the influence of the individual circulation status and scanning times, the ID values of lung lesions were normalized to that of the aorta in the aortopulmonary window level to calculate the NID: NID = ID/ID_aorta_. The slope of the spectral curve was defined as the difference of the CT value at 40 and 60 keV divided by the energy difference (60 − 40) according to the following formula: λ = [CT_(40)_ − CT_(60)_]/(60 − 40).

### Histochemical examination

Tumor specimens were analyzed by a pathologist with 14 years of immunohistochemical staining experience. Without knowing clinical information and spectral CT results, the pathologist numbered and evaluated all sections and analyzed and recorded their pathological types. The histologic criteria used for diagnosis were in accordance with the World Health Organization Classification of Lung Tumors (12, 13).

### Statistical analysis

Kappa and Intraclass correlation coefficient (ICC) tests were used to test the consistency of the measurement results of the two radiologists, with Kappa > 0.8 or ICC > 0.7 indicating satisfaction. All parameters were finally averaged over the measurements made by the two physicians. The normal distribution of all measured data was tested by the Shapiro–Wilk test. Measurements that follow a normal distribution were expressed as mean (standard deviation). In addition, the counting data were expressed by n (%). Independent sample t-test was adopted for all measurement parameters, and the chi-square test was adopted for counting data. For parameters that were significantly different, the results of the pathological examination were used as the gold standard, receiver operating characteristic (ROC) curves were drawn, and the diagnostic efficiency of each parameter is analyzed. First, univariate analysis regression model was established to analyze the correlation between morphological parameters and SC and AC. Then, multivariate logistic regression analyses were applied to identify independent predictors of SC or AC, with the model of CT features alone and CT features combined with optimal spectral parameters; factors with *p* < 0.05 on univariate analysis were used as the input variables for multiple logistic regression analysis, with the final model selected with the forward selection; model I (CT morphological parameters), model II (optimal spectral parameters), model III (morphological parameters and CT enhancement value), and model IV (morphological parameters and optimal spectral parameters) were established respectively. Finally, the ROC curves of the above models are drawn, and their diagnostic efficiency is analyzed. In the case of two-tailed *p*-value < 0.05, the difference was considered statistically significant.

All data were analyzed using SPSS 21.0.0 (IBM Corp., Armonk, NY, USA), and graphics were drawn using GraphPad Prism version 9.0.2 (GraphPad software Inc., San Diego, USA).

We conducted an *a priori* power analysis to test the adequacy of our sample size to independent sample t-test using G*Power (26). We specified an alpha level of 0.05, a 1-β error probability of 0.80, and an effect size (f = 0.50) for an estimated medium effect. The results of the analysis suggested a total recommended sample size of 128. A *post-hoc* power analysis revealed that a sample size of 197 (lung AC : SC = 27:70) resulted in a reported power of 0.917 to detect a medium effect (f = 0.50) with an alpha level of 0.05.

## Results

### Patients’ information

Eleven patients with poor image quality, 45 patients with lymph nodes and/or distant metastases, 32 patients with focal necrosis greater than one-half of the maximum cross-sectional area, and 60 patients without pathological findings or with previous treatment were excluded. Finally, a total of 197 patients met the inclusion criteria, including 70 patients with SC (male patients, 91.4%) and 127 patients with AC patients (male patients, 52.8%). The clinical data are shown in **Table 1**.

**Table 1:** Patient characteristics.

Variable	AC	SC	Statistic	*P*-value
No. of patients	127	70		
Age, mean (SD)	63.8 (9.5)	62.5 (8.4)	−0.969	0.334
Man	64.4 (9.0)	63.2 (8.1)		
Woman	63.3 (10.2)	55.0 (8.2)		
Sex, n (%)			30.294	<0.001
Male	67 (52.8)	64 (91.4)		
Female	60 (47.2)	6 (8.6)		
Diagnostic technique, n (%)			9.164	0.010
Transbronchial biopsy	11 (8.7)	17 (24.3)		
CT-guided biopsy	49 (38.6)	24 (34.3)		
Surgery	67 (52.8)	29 (41.4)		

AC, adenocarcinoma; SC, squamous cell carcinoma.

### CT characteristics of the SC and AC

Univariate analysis revealed that seven CT features differed significantly between SC and AC in **Table 2**: tumor location (distribution), lobulation, spicule, air bronchogram, vacuole sign, lung atelectasis and/or obstructive pneumonia, and vascular involvement (all *p* < 0.05). AC was more frequently found in the periphery, and the lobulation, spicule, air bronchogram, and vacuole sign were more likely to be observed among AC. Tumors with lung atelectasis and/or obstructive pneumonia and vascular involvement were more likely to be observed among SC. When it came to other CT features, no significant differences were noted between AC and SC. **Table 3** shows the traditional CT quantitative parameters, and there was no significant difference in CT values during plain scan. AP-CT value of AC was higher than that of SC, and AP-enhancement value of AC was higher than that of SC. The VP-CT value of AC was higher than that of SC, and the VP-enhancement value of AC was higher than that of SC.

**Table 2:** Morphological parameters in patients with AC and SC.

Characteristics	AC	SC	*P*-value	Univariate OR (95% CI)	*P*-value	Multivariable OR (95% CI)
**Location, n (%)**						
Distribution			**<0.001**		**0.007**	
Peripheral	83 (65.4)	12 (17.1)		9.117 (4.433, 18.751)		4.370 (1.485-12.859)
Central	44 (34.6)	58 (89.2)		Reference		Reference
**Lobe location**						
Right upper lobe	27 (21.3)	10 (14.3)	0.525	1.440 (0.468, 4.430)		
Right middle lobe	33 (26.0)	17 (24.3)	0.948	1.035 (0.366, 2.925)		
Right lower lobe	23 (18.1)	16 (22.9)	0.626	0.767 (0.263, 2.234)		
Left upper lobe	29 (22.8)	19 (27.1)	0.697	0.814 (0.289, 2.291)		
Left lower lobe	15(11.8)	8 (11.4)		Reference		
**Diameter, mean (SD)**						
Long-axis diameter (cm)	4.28 (1.67)	4.73 (1.92)	0.087	0.867 (0.736, 1.021)		
Short-axis diameter (cm)	3.00 (1.55)	3.19 (1.31)	0.394	0.918 (0.755, 1.117)		
**Margin, n (%)**						
Contour (Irregular)	59 (46.5)	41 (58.6)	0.105	0.614 (0.340, 1.107)		
Border definition (poorly)	65 (51.2)	46 (65.7)				
Lobulation (Y)	76 (59.8)	30 (42.9)	**0.023**	1.987 (1.100, 3.590)	0.528	1.406 (0.448–4.049)
Spicule (Y)	71 (55.9)	25 (35.7)	**0.007**	2.282 (1.251, 4.164)	0.198	2.037 (0.690–6.017)
**Internal, n (%)**						
Air bronchogram (Y)	73 (57.5)	22 (31.4)	**0.001**	2.949 (1.594, 5.456)	**0.004**	5.339 (1.729–16.484)
Vacuole sign (Y)	28 (22.0)	3 (4.3)	**0.003**	6.316 (1.846, 21.618)	**0.047**	7.330 (1.030–52.184)
Vessel convergence (Y)	86 (67.7)	44 (62.9)	0.491	1.239 (0.673, 2.284)		
Liquefactive necrosis (Y)	51 (40.2)	36 (51.4)	0.128	0.643 (0.352, 1.141)		
Calcification (Y)	15 (11.8)	15 (21.4)	0.076	0.491 (0.224, 1.077)		
**Other, n (%)**						
Pleural indentation (Y)	54 (42.5)	23 (32.9)	0.185	1.512 (0.821, 2.783)		
Mediastinal/hilar lymphadenopathy (Y)	60 (47.2)	25 (35.7)	0.119	1.612 (0.884, 2.938)		
Lung atelectasis and/or obstructive pneumonia (Y)	11 (8.7)	16 (22.9)	**0.007**	0.320 (0.139, 0.736)	0.536	1.778 (0.287–11.003)
Vascular involvement (Y)	12 (9.4)	22 (31.4)	**<0.001**	0.228 (0.104, 0.497)	**0.047**	5.121 (1.018–25.749)

AC, adenocarcinoma; SC, squamous cell carcinoma; Y, yes.

Values in bold indicate statistical significance, p < 0.05.

**Table 3:** Conventional CT enhancement values and spectral parameters in patients with AC and SC.

	AC	SC	t-value	*P*-value
**Plain CT value, mean (SD)**	34.12 (12.75)	30.07 (13.10)	0.545	0.586
**AP, mean (SD)**				
AP-CT value (Hu)	76.37 (12.17)	67.43 (11.67)	5.006	<0.001
AP-enhancement value (Hu)	40.44 (9.02)	34.36 (15.22)	3.520	<0.001
Zeff	8.07 (0.23)	7.85 (0.16)	8.024	<0.001
ID	1.41 (0.47)	0.94 (0.28)	8.683	<0.001
NID	0.13 (0.04)	0.09 (0.03)	5.482	<0.001
λ	3.42 (1.10)	2.33 (0.69)	8.579	<0.001
**VP, mean (SD)**				
VP-CT value (Hu)	87.47 (14.60)	75.03 (11.55)	6.148	<0.001
VP-enhancement value (Hu)	51.54 (12.94)	41.95 (16.51)	4.504	<0.001
Zeff	8.26 (0.23)	7.96 (0.16)	10.962	<0.001
ID	1.78 (0.51)	1.16 (0.30)	10.859	<0.001
NID	0.39 (0.13)	0.29 (0.08)	7.354	<0.001
λ	4.42 (1.28)	2.85 (0.72)	11.009	<0.001

AC, adenocarcinoma; SC, squamous cell carcinoma; AP, arterial phase; VP, venous phase; Zeff, effective atomic number; ID, iodine density; NID, normalized iodine density; λ, the slope of spectral curve.

### CT spectral quantitative parameters of the SC and AC

As shown in **Table 3**, all CT spectral parameters in the AC group in both the arterial and venous phases were higher than in the SC group (P<0.001).

### Diagnostic implication

**Figure 2:**
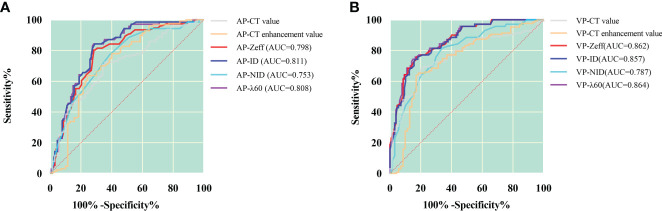
ROC curves for dual-layer spectral detector CT quantitative parameters between squamous cell carcinoma and adenocarcinoma in the arterial phase **(A)** and the venous phase **(B)**. In the arterial phase **(A)**, the ID has the best diagnostic efficiency; AUC, 0.811; sensitivity and specificity, 71.7% and 82.9%, respectively; in the venous phase **(B)**, the λ has the best diagnostic efficiency; AUC, 0.864; sensitivity and specificity, 85.8% and 74.3%, respectively. AP, arterial phase; VP, venous phase; Zeff, effective atomic number; ID, iodine density; NID, normalized iodine density; λ, slope of 40- to 60-keV spectral curve.

Because spectral quantitative parameters have statistically significant differences between SC and AC in the AP and the VP, diagnostic capacity was assessed using ROC curves in **Table 4** and **Figure 2**. The spectral parameters in VP are generally better than those in AP in differentiating SC from AC. The VP-λ has the best diagnostic efficacy, with AUC = 0.864, 95% CI = 0.813~0.915, sensitivity = 85.8%, and specificity = 74.3%.

**Table 4:** Performance of differential parameters in distinguishing the AC and SC in the receiver operating characteristic analysis.

Parameter	AUC	Thresholds	Sensitivity (%)	Specificity (%)	95% CI	*P-*value
**AP**						
CT value	0.704	69.47	72.4	64.3	0.692~0.779	<0.001
CT enhancement value	0.721	36.50	63.8	75.7	0.641~0.802	<0.001
Zeff	0.798	7.975	71.7	82.9	0.735~0.860	<0.001
ID	0.811	1.145	71.7	82.9	0.737~0.864	<0.001
NID	0.753	0.110	68.5	74.3	0.683~0.823	<0.001
λ	0.808	2.800	71.7	84.3	0.747~0.869	<0.001
**VP**						
CT value	0.760	82.13	69.3	78.6	0.691~0.828	<0.001
CT enhancement value	0.733	48.65	65.4	81.4	0.656~0.811	<0.001
Zeff	0.862	8.055	82.7	75.7	0.810~0.913	<0.001
ID	0.857	1.265	83.5	74.3	0.805~0.909	<0.001
NID	0.787	0.297	79.5	65.7	0.721~0.852	<0.001
λ	0.864	3.088	85.8	74.3	0.813~0.915	<0.001

AP, arterial phase; VP, venous phase; Zeff, effective atomic number; ID, iodine density; NID, normalized iodine density; λ, the slope of spectral curve.

### Multivariable analysis and joint diagnostic efficiency

**Figure 3:**
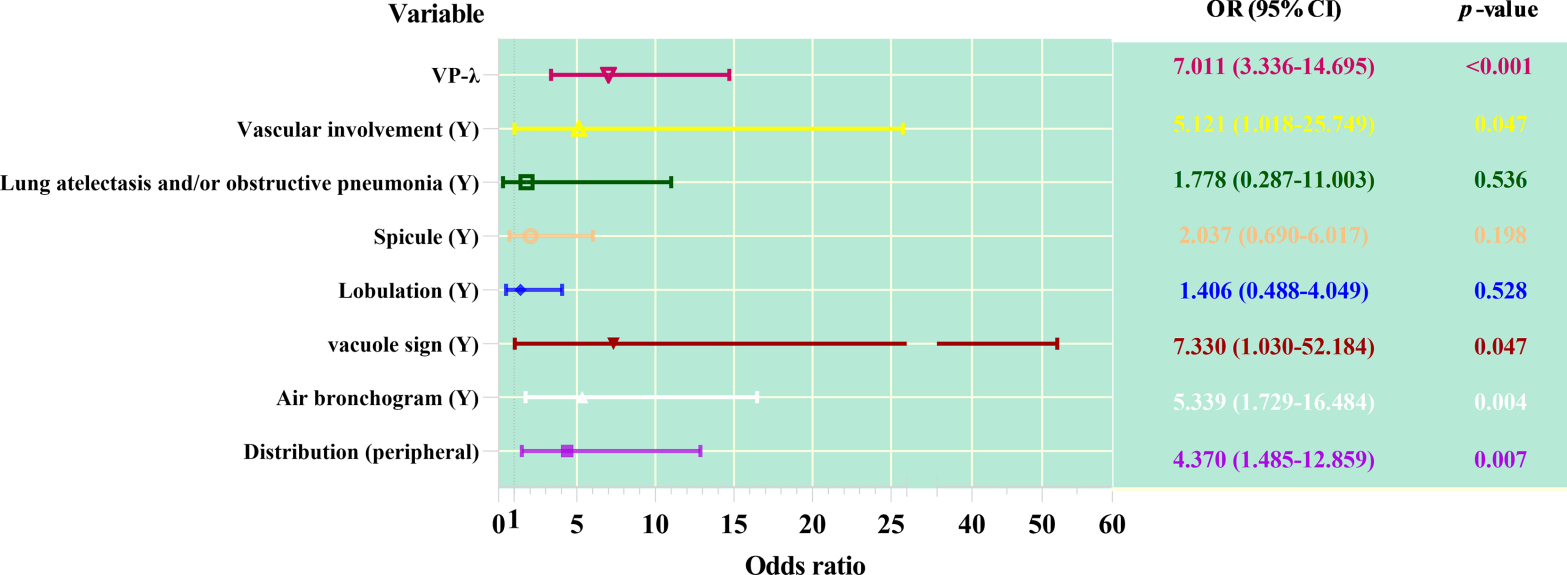
Forest plot of multivariable regression analysis of dual-layer spectral detector CT metrics combined with CT features predicting adenocarcinoma from squamous cell carcinoma.

In multivariate analysis, after adjusting for other confounding factors, models of distribution, air bronchus, vacuole sign, and VP-λ are associated with AC (**Figure 3**).

**Figure 4:**
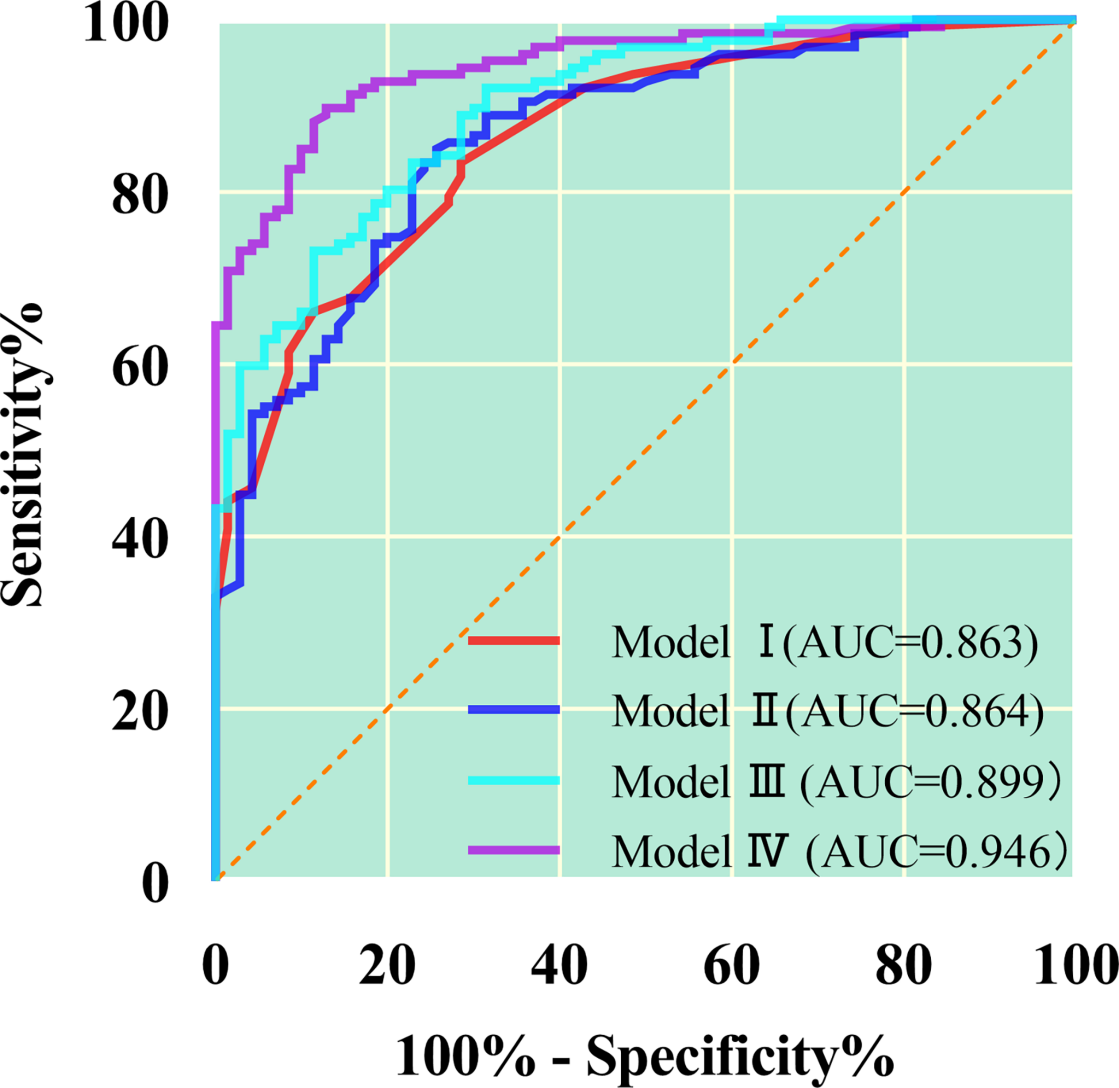
The quantitative parameters of different models were used to identify the lung adenocarcinoma from squamous cell carcinoma. The combination of morphology and λ in the venous phase has the greatest diagnostic efficiency; AUC, 0.946; sensitivity and specificity, 89.8% and 87.1%, respectively. Model I, morphological parameters; Model II;, λ in the venous phase; Model III, combination of morphology and CT value in the venous phase; Model IV, combination of morphology and λ in the venous phase.

As shown in **Table 5** and **Figure 4**, ROC curve analysis in diagnosing of the AC and SC using morphological parameters, optimum spectral parameter (VP-λ), combining morphological parameters with CT value, and combining morphological parameters with spectral parameters in the VP. When morphological parameters were combined with VP-λ, the diagnostic efficiency is the highest, with AUC = 0.946, 95% CI = 0.917–0.975, sensitivity = 89.8%, and specificity = 87.1%.

**Table 5:** Different models in distinguishing the AC and SC in the receiver operating characteristic analysis.

Parameter	AUC	Thresholds	Sensitivity (%)	Specificity (%)	95% CI	*P-*value
Model I	0.863	—	83.5	71.4	0.813~0.914	<0.001
Model II	0.864	3.088	85.8	74.3	0.813~0.915	<0.001
Model III	0.899	—	73.2	88.6	0.857~0.941	<0.001
Model IV	0.946	—	89.8	87.1	0.917~0.975	<0.001

Model I, morphological parameters; Model II, λ in the venous phase; Model III, combination of morphology and CT value in the venous phase; Model IV, combination of morphology and λ in the venous phase.

**Figure 5:**
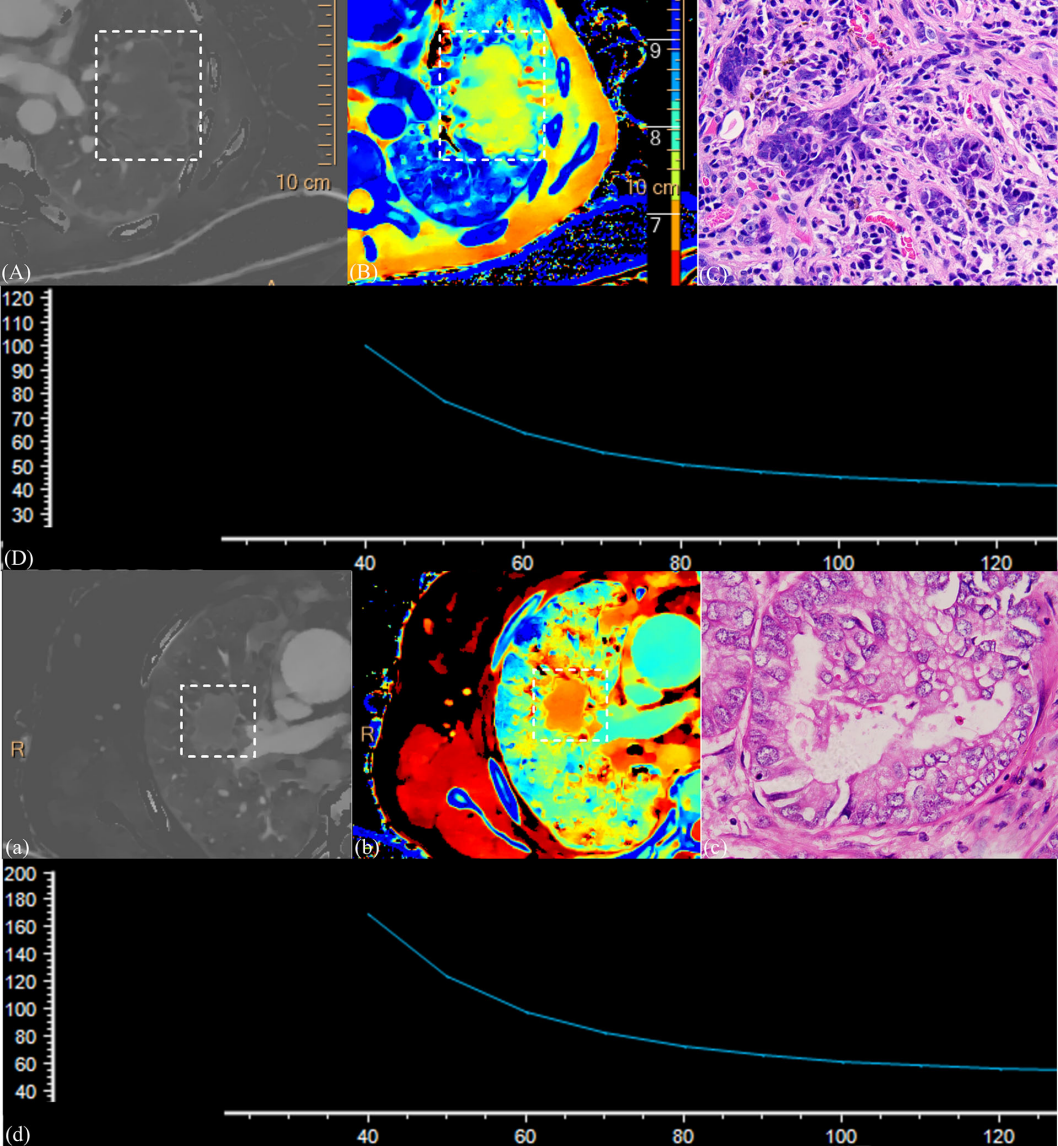
(1) CT images and the pathological section of a 58-year-old man with squamous cell carcinoma in the venous phase **(A–D)** and a 63-year-old man with adenocarcinoma in the venous phase **(a–d)**. Iodine density images **(A, a)**; Z-effective images **(B, b)**; the dashed box point to lung cancer tissue. Magnification: C, ×600; slope of spectral curve in the arterial phase; the horizontal axis represents the energy (keV), and the vertical axis represents mean attenuation (Hu) **(D, d)**. λ = 1.72, ID = 0.70, NID = 0.19, and Zeff = 7.71 for squamous cell carcinoma, and λ = 3.91, ID = 1.59, NID = 0.31, and Zeff = 8.19 for adenocarcinoma. Significant differences were observed in all parameters representing these cases.

**Figure 5** shows the imaging and pathological images.

### Reliability of measurements

For all the given parameters, the Kappa and ICC tests found that the measurement results were found to be consistent between the two radiologists (*p* > 0.05).

## Discussion

Our results showed that the spectral parameters of the VP have a higher diagnostic value in differentiating SC from AC than those of the AP. Morphological parameters also have important diagnostic value, in which distribution, air bronchus, and vacuole sign are associated with AC. The joint diagnosis efficacy is higher than that of single index, and the joint diagnosis of spectral parameters and morphological parameters had the best efficacy. Therefore, the combined diagnosis of energy spectral parameters and morphological parameters is more effective and instructive for the pathological classification of lung cancer.

CT is widely used in the diagnosis of lung diseases. In recent years, spectral CT has become a hot spot in the study of lung cancer. However, in diagnostic studies of lung cancer (8–11), most researchers were keen to study the optimal diagnostic performance of quantitative parameters, ignoring the morphological features. Therefore, we have supplemented the study of the morphological features of tumors. Our results suggest that the morphological features of tumors can be used for histological inference of AC and SC, although some of the signs are not significant. Several previous studies have compared the clinical and imaging features of lung cancer of different pathological types. Chu et al. (14) showed that the radiologic and microscopic findings correlate well with each other and are closely associated with tumour prognosis. Understanding their morphological features is helpful for early identification and diagnosis. Wang et al. (15) have shown that AC and SC have different clinical and imaging characteristics and that imaging features are useful for differential diagnosis. Koenigkam et al. (16) found that SC more likely to occur in male patients, more frequently found in the central distribution, and less lobulation, burr, and air bronchogram. In addition, previous studies did not analyze the contrast enhancement between AC and SC, but we did a detailed analysis. We found the differences between them and verified the diagnostic value of morphological features.

Conventional CT is used to differentiate subtypes of tumors by their size, shape, and attenuation value. Although it has some advantages, the accuracy of diagnosis is still limited (11, 17). The spectral parameters provide quantitative analysis for histological diagnosis of NSCLC, and its accuracy is higher than that of conventional CT, making up for the deficiency of conventional CT. There are several reasons: i. From the perspective of histology, AC is a malignant epithelial neoplasm of glandular differentiation, in which glandular tubular and glandular luminal–like structures are common, and it contains more microvessels with abundant blood supply (18). Whereas, SC is defined as keratinized malignant epithelial tumor, which mostly grows in a stacked pattern, and its internal structure is dense, with frequent tumor nests, keratinized beads, and intercellular bridges, and the internal part is mostly accompanied by liquefactive necrosis (18, 19). ii. The degree of tumor enhancement depends only on the amount of contrast medium in the tumor vessels. The central type of lung cancer is mainly SC, mainly supplied by bronchial artery, whereas the peripheral type of lung cancer is mainly AC with dual blood supply of bronchial artery and pulmonary artery (20, 21). Because the blood supply from the bronchial artery is later than that from the pulmonary artery, the VP better reflects the blood supply of the tumor.

ID reflects intravascular blood flow distribution and vascular status by quantitative analysis of iodine content, but it is influenced by many factors, including cardiac output and blood volume of the patient, concentration and flow rate of the contrast medium, and dose and rate of injection (20). NID was defined as the ratio of tumor ID to that of the aorta or subclavian arteries at the same level, and most researchers (8, 21–23) state that NID can reduce the effect of individual circulatory variability on tumor iodine content and thus more accurately reflect the blood supply of the lesion. However, in this comparison of AC and SC, we found that ID is more effective than NID in both the AP and the VP, which is consistent with the results of the study by Li et al. (10). This suggests that NID depends on the extent of lesion and aortic enhancement, and changes in aortic ID may cause NID to deviate.

Energy spectrum curve is the curve of material decay varying with x-ray energy, which reflects the decay characteristics of material. Theoretically, each substance has its own specific spectrum curve, and the slope of the spectrum curve decreases with the expansion of its range (24). The K edge of iodine is 33.2 keV, so the lower the keV, the higher the CT value (25). From IQon, monochrome images of the 40- to 200-keV energy range can be obtained. However, not all images can be used to observe and diagnose lesions, because image quality varies at different energy levels. Low–kilo–electron volt images can improve tissue enhancement, increase tissue contrast, and make the display of small lesions clearer. Equivalent kilo–electron volt images can reduce image noise and improve image quality. This study shows that VP-λ is conducive to the differentiation of lung SC and AC. Jia et al. (9) reported that the spectral parameters of SC in the AP and the VP were larger than those of AC, and this is different from our experimental results.

Zeff is the atomic number of an element that has the same decay coefficient as a compound or mixture and can be used to identify the tissue composition of a substance, especially in substances with similar CT values. It is a quantitative indicator of different substances (11, 26). Moreover, Zeff can indirectly provide information about the accumulation of contrast media (27). Previous studies have shown that Zeff can identify substances (28, 29). In the differential diagnosis between AC and SC, Zeff in VP was greater than that in AP. This is related to the blood supply of the tumors described above, their histological characteristics, the growth pattern of the tumors, and the changes of their surrounding microenvironment.

In this study, we provide not only quantitative parameters for tumor enhancement but also qualitative parameters for morphological features. The diagnostic ability of combining morphological features with quantitative parameters, especially DLCT, has been significantly improved, which confirms the additional diagnostic value of quantitative analysis of spectral parameters. Although there are more and more studies on quantitative data, CT morphology of lung cancer remains an important indicator of cancer diagnosis. On the one hand, they are closely related to the growth characteristics of tumors and easy to collect. On the other hand, in the era of precision medicine and big data, a single spectral parameter may also be considered unreliable.

The limitations of this study are as follows: (1) because of the small sample size, this study failed to stratify the degree of differentiation of tumors; (2) unenhanced spectral parameters were not included in the study; (3) not all pathological sections were matched with imaging ROI; and (4) some patients’ unenhanced images were replaced by virtual unenhanced images, which may affect our results.

In summary, the combination of morphological features and DLCT spectral parameters improved the diagnostic efficiency in distinguishing SC from AC. This may help clinicians develop initial treatment strategy and prognostic predictions. Although relatively accurate pathological diagnosis can be obtained by invasive methods, complications or patient intolerance still exist.

## Data availability statement

The raw data supporting the conclusions of this article will be made available by the authors, without undue reservation.

## Ethics statement

The studies involving human participants were reviewed and approved by Nanxishan Hospital of Guangxi Zhuang Autonomous Region Institution Review Committee [NO.2020(KY-E-30)]. The patients/participants provided their written informed consent to participate in this study. Written informed consent was obtained from the individual(s) for the publication of any potentially identifiable images or data included in this article.

## Author contributions

First author: RM. Co-author: ZM; ZG. Other authors: XQ; GH; XY; HJ; PY; MD; XDZ. Guarantor and correspondent: XQZ. All authors contributed to the article and approved the submitted version.

## Acknowledgments

The authors thank the dedicated this project participants, their loved ones, and the devoted staff and trainees who contributed to recruitment, screening, and enrollment of the cohort.

## Conflict of interest

Author XZ is employed by Philips China Investment Co.

The remaining authors declare that the research was conducted in the absence of any commercial or financial relationships that could be construed as a potential conflict of interest.

## Publisher’s note

All claims expressed in this article are solely those of the authors and do not necessarily represent those of their affiliated organizations, or those of the publisher, the editors and the reviewers. Any product that may be evaluated in this article, or claim that may be made by its manufacturer, is not guaranteed or endorsed by the publisher.
